# Comparison between Conventional and Simple Measuring Methods of Mean Nocturnal Baseline Impedance in Pediatric Gastroesophageal Reflux Disease

**DOI:** 10.3390/clinpract14050134

**Published:** 2024-08-27

**Authors:** Radu Samuel Pop, Lăcrămioara Eliza Chiperi, Vlad-Ionuț Nechita, Sorin Claudiu Man, Dan Lucian Dumitrașcu

**Affiliations:** 1 3rd Department of Pediatrics, “Iuliu Hațieganu” University of Medicine and Pharmacy, 400217 Cluj-Napoca, Romania; claudiu.man@umfcluj.ro; 2 Department of Pediatrics, George Emil Palade University of Medicine, Pharmacy, Sciences and Technology, 540136 Târgu Mureș, Romania; lacramioara-eliza.pop@umfst.ro; 3 Department of Medical Informatics and Biostatistics, “Iuliu Hațieganu” University of Medicine and Pharmacy, 400349 Cluj-Napoca, Romania; nechita.vlad@umfcluj.ro; 4 3rd Pediatric Clinic, Clinical Emergency Hospital for Children, 400217 Cluj-Napoca, Romania; 5 2nd Department of Internal Medicine, “Iuliu Hațieganu” University of Medicine and Pharmacy, 400006 Cluj-Napoca, Romania; ddumitrascu@umfcluj.ro

**Keywords:** GERD, pediatric, impedance-pH monitoring, MNBI

## Abstract

(1) Background: Multichannel intraluminal impedance–pH (MII-pH) monitoring is commonly used to diagnose gastroesophageal reflux disease (GERD). The mean nocturnal baseline impedance (MNBI) is an important parameter, reflecting the esophageal mucosal integrity and improvement in GERD. This study aims to evaluate the correlation between conventionally measured MNBI and a recently described simple MNBI measurement method in diagnosing pediatric GERD. (2) Methods: This prospective observational study enrolled 64 children aged one month to 18 years who underwent 24 h MII-pH monitoring. Conventional MNBI was measured during stable 10 min intervals at night, while the simple MNBI method averaged impedance throughout the nocturnal supine period. (3) Results: Strong correlations were found between conventional and simple MNBI values across all impedance channels in both infants (r > 0.85) and older children (r > 0.9). Conventional and simple MNBIs in the most distal channel (Z6) effectively differentiated non-erosive reflux disease (NERD) from other phenotypes, with AUCs of 0.864 and 0.860, respectively. The simple MNBI demonstrated good diagnostic performance with similar sensitivity and specificity to the conventional MNBI. (4) Conclusions: Including MNBI measurements into routine MII-pH monitoring may enhance GERD diagnosis and reduce the need for more invasive procedures.

## 1. Introduction

Gastroesophageal reflux disease (GERD) represents a common condition among infants and children that is characterized by the reflux of gastric contents into the esophagus, resulting in a variety of symptoms that significantly impact the child’s quality of life or that lead to complications [1,2,3,4].

In infants, the global prevalence of GERD has not been determined [5]. Curien-Chotard et al. reported that infant GERD had a prevalence peak at one month of age (19%) and subsequently decreased from 9% at three months to 2% at 12 months of age. In older children, prevalence has a wide range between 0.2% and 32.0% [6].

Due to its ability to quantify and describe every reflux episode as well as its possible correlation with symptoms, multichannel intraluminal impedance–pH (MII-pH) monitoring has become the most widely used test for diagnosing gastroesophageal reflux disease. In older children, MII-pH can distinguish several GERD phenotypes with different pathophysiologies [7], as follows: Non-erosive reflux disease (NERD) refers to individuals who experience GERD symptoms and who have no proven esophagitis on endoscopy but abnormal acid exposure during MII-pH monitoring [8]. Another two functional phenotypes are reflux hypersensitivity (RH), represented by negative endoscopy, normal acid exposure of the esophagus but positive association between symptoms; and reflux episodes and functional heartburn (FH) in children that can describe typical GERD symptoms and have negative endoscopy, normal esophageal acid exposure and no association between reflux episodes and symptoms during MII-pH monitoring [9,10]. The real challenge is found in patients with inconclusive acid exposure time [11]. In such a scenario, an additional MII-pH parameter is used, namely the number of reflux episodes/24 h, which is considered pathological if it is more than 100/24 h in infants and more than 70/24 h in older children [12,13,14,15].

In recent years, there has been a focus on determining the baseline impedance (BI), which refers to the average impedance of the esophagus when it is empty and when there are no reflux episodes or swallows. Low distal baseline impedance has been observed in patients with esophagitis and proven to negatively correlate with acid reflux metrics [16,17,18,19]. Recently, there has been a proposal to utilize the mean nocturnal baseline impedance (MNBI), which represents the average of three measurements in 10 min stable intervals [20]. MNBI determines the baseline electrical conductivity of the esophageal mucosa when there is no reflux, reflecting the integrity of the esophageal mucosa and the reflux burden [21,22].

The MNBI is considered to improve the diagnosis of GERD [23,24,25]; increase the accuracy in delineating the GERD phenotype, especially in cases of inconclusive acid exposure time [26,27]; and enhance predictions of the anti-reflux treatment response [28,29,30]. Adult protocols have included MNBI as a standard measurement in MII-pH interpretation [11,31]. Additionally, MNBI has been proposed as a marker for laryngopharyngeal reflux, further expanding its diagnostic applications [32].

Nevertheless, the majority of available commercial software does not provide an automatic calculation option, and manually measuring MNBI is time consuming. More time is needed for patients with frequent nocturnal reflux and swallowing. Hoshikawa and al. [33] recently described a new method of MNBI assessment that calculates the average impedance throughout the entire nocturnal supine period. This simple method showed an excellent correlation with the conventional method in the distal esophagus and a moderately good correlation in the proximal esophagus [34].

However, the evaluation of whether this simple method of assessing MNBI is useful in pediatric GERD is not known. This study aimed to evaluate the correlation between conventional MNBI and simply acquired MNBI values and their utility in the diagnosis of GERD non-erosive phenotypes.

## 2. Materials and Methods

### 2.1. Study Design and Patient Selection

We conducted a prospective, observational study between October 2019 and December 2023 at the Emergency Clinical Hospital for Children, 3rd Pediatric Clinic. Patients between one month and 18 years of age referred for MII-pH monitoring were enrolled and were divided into two groups based on age: Group 1, consisting of infants, and Group 2, including children above one year of age. Inclusion criteria for infants were persisting troublesome symptoms suggestive of GERD, and criteria for children > 1 year were represented by negative endoscopy and persistent GERD symptoms despite standard proton-pump inhibitor therapy. The exclusion criteria comprised individuals presenting with eosinophilic esophagitis, psychiatric or neurological impairment, and esophageal motor dysfunctions. Recordings with a duration of less than 20 h were also excluded.

This study was conducted in accordance with the Declaration of Helsinki and was approved by the local ethics committee (Nr. 299/11.09.2019). Written informed consent was also obtained from parents of all the children and infants before their inclusion in the study.

### 2.2. Twenty-Four-Hour Impedance–pH Monitoring

Twenty-four h MII-pH (Sandhill Scientific, Highlands Ranch, CO, USA) monitoring was conducted concurrently in all patients. Age-appropriate catheters were used together with seven impedance sensors and one pH sensor. Each pair of electrodes forms a segment, which creates one impedance channel. Therefore, probes with seven electrodes have a total of six impedance channels (Z1–Z6). Before insertion, calibration was performed using buffer solutions with pHs of 4 and 7. The catheter position was estimated according to the formula proposed by Mutalib et al. [35] and checked through fluoroscopy. Patients fasted for at least 3 h prior to the examination. Acid-suppressive treatment and prokinetic drugs were ceased seven days prior to the investigation, and alginate was stopped 24 h prior, in accordance with the current recommendation [36].

Pathological acid exposure time (AET) was defined as a value of more than 7% in patients older than one year or of more than 10% in infants. According to most guidelines, an AET between 3 and 7% was considered inconclusive [1,13,36]. Conventional parameters were automatically calculated by the Bioview Analysis software: mean acid clearance time (MACT), mean bolus clearance time (MBCT), symptom index (SI), symptom sensitivity index (SSI), and symptom association probability (SAP). Patients older than one year were categorized into several phenotypes: NERD, RH, FH, and indeterminate, with the latter described as having a anormal reflux index not otherwise specified (“normal RI-NOS”) and consisting of patients characterized by normal esophageal acid exposure time and an unreliable symptom association due to fewer than three reported symptoms during MII-pH monitoring, according to Blasi et al. [37]. An SAP was considered reliable when a gastrointestinal symptom was reported at least three times or an extraesophageal symptom was reported at least five times. In patients with an indeterminate AET and a positive SAP and/or SI, the pathological number of reflux episodes was used to differentiate NERD patients from those with RH. Infants were categorized as GERD and normal MII-pH. A manual evaluation of all tracings was also made despite the automatic software analysis provided by Sandhill. The post-reflux swallow-induced peristaltic wave (PSPW) was calculated according to Frazzoni et al. [23].

**Figure 1:**
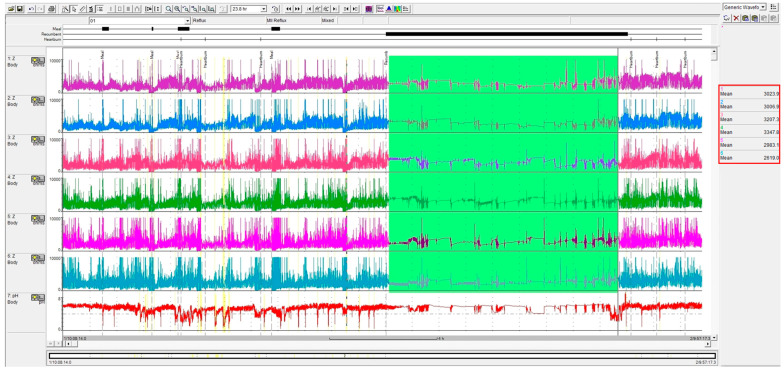
The simple method of measuring the mean nocturnal baseline impedance (24 h multichannel intraluminal impedance tracing, with the nocturnal recumbent period highlighted in green). Mean measurements in each channel using the software’s “electronic ruler” are highlighted in the red box.

The MNBI was measured in every impedance channel (Z1–Z6) by two methods. The conventional MNBI was measured during the night while patients were in a recumbent position, as described by Martinucci et al. [38]. The MNBI was represented by the mean value of 3 measurements of during stable timeframes of 10 min at 1:00 AM, 2:00 AM, and 3:00 AM, excluding pH drops, swallows, or reflux episodes. The simple method of measuring MNBI was also assessed throughout the entire recumbent duration utilizing the software’s “electronic ruler” (Figure 1). If there were two or more supine period markings, we selected the longest one, as described by Hoshikawa et al. [33].

### 2.3. Statistical Methods

Statistical Package for Social Sciences (IBM SPSS^®^ version 29.0.2.0 for Windows, IBM Crop. Armonk, New York, NY, USA) and R version 4.0.5 with R Commander (R Foundation for Statistical Computing, Vienna, Austria) were used for the statistical analysis. The normal quantitative data distribution was evaluated using the Shapiro–Wilk test, skewness, and kurtosis. The mean ± standard deviation or median and interquartile range were used to present normal distributed and non-normal distributed data, respectively. To compare frequencies, we used the chi-square test, and where frequencies were under 5, we used Fisher’s exact test. To compare quantitative data, we used ANOVA analysis, the Mann–Whitney U test, the Kruskal–Wallis test, and the student’s t-test, depending on the distribution. Post hoc analysis was performed using the Games–Howell method for unequal variances and the Tukey method for equal variances. To evaluate the correlation between quantitative data, we used Pearson’s or Spearman’s correlation coefficients. The diagnostic performance of parameters such as the MNBI was evaluated by the area under the receiver operating characteristic (ROC) curve (AUC) analysis using the maximum Youden index, achieving cut-off values for sensitivity and specificity. We considered a *p*-value of less than 0.05 to be statistically significant.

## 3. Results

### 3.1. Patient Characteristics and pH–Impedance Measurements

Seventy patients were eligible for the study. Six children were excluded due to several factors: misplacement of the catheter, the discovery of other diagnoses, or technical issues. Of the sixty-four pediatric patients that were enrolled in the study, 23% were infants and 77% were children. Children categorized into non-erosive phenotypes included 12 children (24.5%) having non-erosive reflux disease (NERD), 20 (40.8%) with reflux hypersensitivity (RH), 13 (26.5%) with functional heartburn, and 4 (8.2%) with normal reflux index not otherwise specified (RI-NOS), as previously published in our pilot study [39]. A total of 67% of the infants were diagnosed with GERD based on the MII-pH results. General characteristics of the patients are presented in Table 1. Our study included 23 male patients (46.9.6%) and 26 female patients (53.1%). There were significantly more male patients having NERD and normal RI-NOS. No difference was observed regarding the BMI percentile among the phenotypes in children, and there was no difference regarding the weight-for-length among infants with GERD compared to those with a normal MII-pH tracing.

Among the infants (group 1), GERD patients had significantly lower PSPW index values compared with those without GERD. There was no significant difference regarding the total number of reflux episodes, proximal extension episodes, bolus clearance time, acid clearance time, and the MNBI in different channels.

Among the children (group 2), NERD patients had a significantly higher number of reflux episodes and a higher number of proximal reflux episodes compared with non-NERD patients (*p* = 0.003 and *p* = 0.001, respectively). There was no statistically significant difference between infants with GERD and normal MII-pH regarding the number of reflux episodes or bolus and acid clearance time. NERD patients had a significantly lower PSPW index value compared with non-NERD patients. We found significantly higher PSPW index values among patients with FH compared with those with RH (*p* < 0.01).

### 3.2. Conventional MNBI Measurement

There was a significant statistical difference between conventional MNBI-Z5 and MNBI-Z6 values among GERD phenotypes (*p* = 0.001), as shown in Table 1. Patients diagnosed with NERD presented the lowest values, which were significantly different from patients with normal acid exposure (*p* < 0.001). Patients with RH had a significantly lower value of conventional MNBI-Z6 (*p* = 0.03) when compared with patients with FH. FH patients had significantly higher conventional MNBI-Z5 values (*p* = 0.002) and conventional MNBI-Z6 values (*p* < 0.001) compared with non-FH patients.

### 3.3. Simple MNBI Measurement

**Figure 2:**
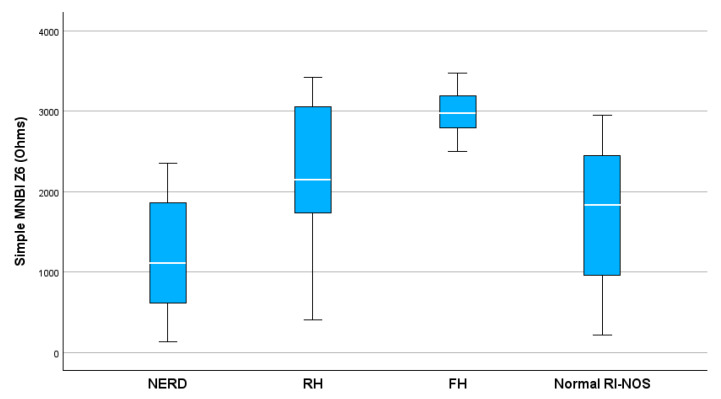
Distribution of simple MNBI-Z6 values between GERD phenotypes (*p* < 0.001). MNBI, mean nocturnal baseline impedance; NERD, non-erosive reflux disease; RH, reflux hypersensitivity; FH, functional heartburn; Normal RI-NOS, normal reflux index not otherwise-specified; Z6, the most distal impedance channel.

The distribution of simple MNBI-Z6 values is illustrated in Figure 2. The simple MNBI measurements in all impedance channels among the different subgroups are shown in Table 2. We found significant statistical differences comparing MNBI-Z5 (0.005) and MNBI-Z6 values (<0.001) among the GERD phenotypes. NERD patients had the lowest MNBI-Z5 and MNBI-Z6 values, which were significantly different from patients with normal acid exposure (*p* = 0.006 and *p* < 0.001, respectively). Patients with RH showed significantly lower values of simple MNBI-Z6 (*p* = 0.02) compared with those with FH. FH patients had significantly higher simple MNBI-Z5 values (*p* = 0.005) and simple MNBI-Z6 values (*p* < 0.001) compared with non-FH patients. There was no significant difference between simple MNBI values among all the impedance channels between infants with GERD and those with a normal MII-pH.

### 3.4. Comparison between Conventional and Simple MNBI Measurements

We found similar values of MNBI in every impedance channel using the two methods of measurement, except for MNBI-Z4, as presented in Table 3 and Table 4. We found a strong correlation between simple MNBI and conventional MNBI in all channels in children (r > 0.9, *p* < 0.001) as well as in infants (r > 0.85, *p* < 0.001). We also found a strong inverse correlation between conventional MNBI-Z6 and AET (r = −0.528, *p* < 0.001), as well as between simple MNBI-Z6 and AET (r = −0.519, *p* < 0.001).

We found a very strong correlation between simple MNBI and conventional MNBI values in MNBI-Z5 in all GERD phenotypes except those categorized as normal RI-NOS. A strong correlation was found between simple MNBI and conventional MNBI values when measuring in the most distal channel (Z6) in all phenotypes, as shown in Table 5.

**Figure 3:**
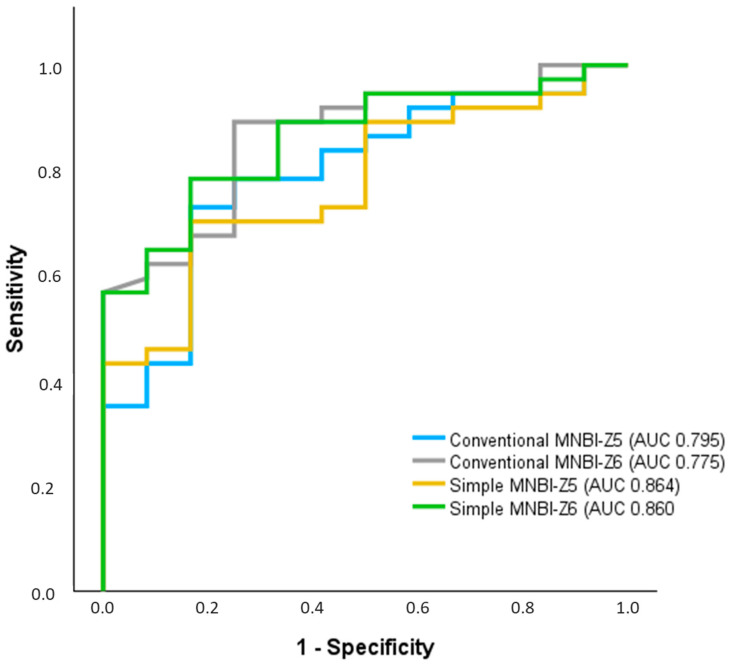
ROC curves for conventional mean nocturnal baseline impedance in MNBI-Z5, conventional MNBI-Z6, simple MNBI-Z5, and simple MNBI-Z6 in differentiating NERD patients from non-NERD patients.

Using an ROC curve analysis (Figure 3), we observed a good overall performance of both conventional MNBI-Z5 (AUC 0.795, CI 95% 0.656–0.934) and simple MNBI-Z5 (AUC 0.775, CI 95% 0.637–0.912) to differentiate between NERD and non-NERD phenotypes. Conventional MNBI-Z5 was able to distinguish NERD from non-NERD phenotypes with a sensitivity of 72.97% and a specificity of 83.33% at a cut-off value of 2329 Ω. Simple MNBI-Z5 was able to distinguish NERD from non-NERD phenotypes with a sensitivity of 70.27% and a specificity of 83.33% at a cut-off value of 2404 Ω.

Conventional MNBI-Z6 (AUC 0.864, CI 95% 0.756–0.972) and simple MNBI-Z6 (AUC 0.860, CI 95%, 0.754–0.967) showed similar overall performance in distinguishing between NERD and non-NERD children at a cut-off value of 1935 Ω, while simple MNBI-Z6 diagnosed NERD with a sensitivity of 78.38% and specificity of 83.33%. Using a DeLong test, we found no significant difference between the performance of simple and conventional MNBI-Z5 or MNBI Z6 in distinguishing NERD patients from those with normal acid exposure.

**Figure 4:**
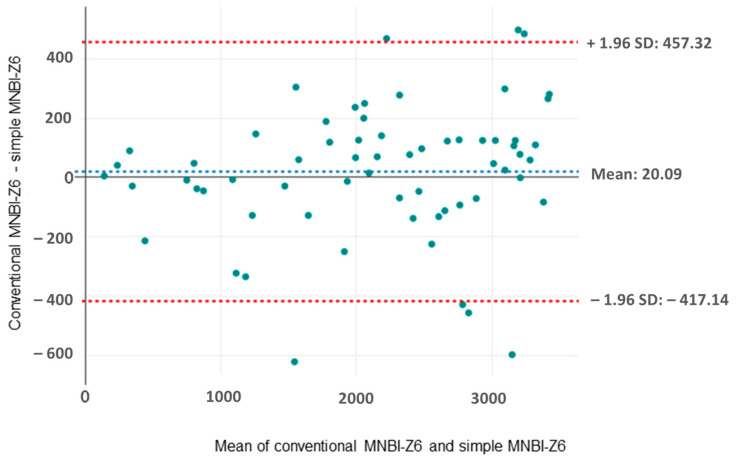
Bland–Altman plot for conventional and simple mean nocturnal baseline impedance measurements in the most distal channel. MNBI-Z6, mean nocturnal baseline impedance in channel Z6; SD, standard deviation.

The Bland-Altman plot for MNBI-Z6 (Figure 4) displayed a uniform distribution of points, demonstrating a strong agreement between the two methods and indicating minimal bias when using either the simple or conventional methods in this channel.

## 4. Discussion

Studies have indicated that the presence of acid can negatively impact the intercellular junctional complexes in the esophageal epithelium, causing leakage between cells and the dilated intercellular spaces [40,41,42,43] The injured epithelium persistently exposed to acid has been demonstrated to have a decreased baseline impedance, both in adults and children [16,17,18,19]. We found a significant strong correlation between conventional MNBI in the most distal channel and acid exposure time (r = −0.528, *p* < 0.001), as well as between simple MNBI-Z6 and AET (r = −0.519, *p* < 0.001). This is consistent with several studies that showed that pediatric patients with abnormal acid exposure have significantly lower MNBI values compared with those with normal acid exposure times. Blasi et al. [37] and Rosado-Arias et al. [44] described children with severe esophagitis as having a lower MNBI than patients without severe esophagitis.

A study conducted by Eiamkulbutr et al. [45] reported that the MNBI could diagnose GERD in children with a sensitivity of 50.0% and a specificity of 33.33% at a cutoff value of 1466 Ω. Rosado-Arias et al. [46] showed that MNBI in the most distal channel had an acceptable ability to diagnose GERD in children with a sensitivity of 100% and a specificity of 45% at a cutoff value of 2183 Ω. This indicates that the assessment of nocturnal baseline impedance might be a useful diagnostic method, perhaps having the potential to eliminate the need for upper gastrointestinal endoscopy in the diagnosis of esophagitis.

We found significantly lower conventional MNBI-Z6 and simple MNBI-Z6 values in NERD patients compared with those with normal acid exposure, which is in concordance with the data reported by Blasi et al. [37]. Using an ROC curve analysis, we observed an excellent ability of simple MNBI in the most distal channel (AUC 0.864, CI 95% 0.756–0.972) to differentiate between NERD and non-NERD phenotypes. Simple MNBI-Z6 was able to differentiate NERD from non-NERD phenotypes with a sensitivity of 78.38% and specificity of 83.33% at a cut-off value of 1935 Ω.

Conventional MNBI-Z6 and simple MNBI-Z6 showed an excellent ability to discriminate FH from the rest of the phenotypes. Conventional MNBI-Z6 (AUC 0.802, CI 95% 0.676–0.928) was able to diagnose FH with a sensitivity of 84.6% and a specificity of 80.6% at a cut-off value of 2563. However, simple MNBI-Z6 cm was better able to predict the presence of FH (AUC 0.823, CI 95% 0.697–0.949), with a sensitivity of 92.3% and a specificity of 80.6% at a cut-off value of 2494 Ω. We found a very strong correlation between simple MNBI-Z6 and MNBI-Z7 in NERD children (r = 0.953, *p* < 0.001), showing the potential of simple MNBI measurement to be used in standard impedance assessment.

Both conventional MNBI-Z6 and simple MNBI-Z6 demonstrated the same ability to differentiate between NERD and RH in patients with an inconclusive reflux index (AUC = 0.741). Conventional MNBI-Z6 was able to diagnose NERD with a sensitivity of 100% and a specificity of 55.67% at a cut-off value of 1447 Ω. In adult populations, MNBI has been demonstrated to be useful in distinguishing between GERD phenotypes, especially when conventional metrics provide inconclusive results [26,30,33,47,48].

Due to its utility, it may be used to diagnose GERD without the need for endoscopy, offering the advantage of being less invasive and not requiring fasting or sedation.

We found no difference in conventional MNBI or simple MNBI values in infants with GERD compared to those without GERD in all impedance channels. This could be explained by the predominance of non-acid reflux episodes in this age group. Several studies have shown that in this age group, non-acid or weakly acid reflux episodes are predominant [49,50,51,52,53]. The utility of the simple method to measure MNBI is debatable due to the fact that infants have meals during the night and also have more reflux episodes.

To the best of our knowledge, there are no published studies regarding the validation of the simple MNBI measurement method in children. We found a strong correlation between simple MNBI and conventional MNBI in all channels in children (r > 0.9, *p* < 0.001), especially in the distal esophagus, as well as in infants (r > 0.85, *p* < 0.001). This is consistent with the data published in adult populations by Hoshikawa et al. [33], who found a very strong correlation between conventional MNBI and simple MNBI in the most distal channels (r = 0.95, *p* < 0.001). Another study conducted by Park et al. [34] showed a very good correlation as well between the two measurement methods in the distal esophagus (r = 0.94, *p* < 0.001) in adults.

Values obtained by the two methods of measurement showed very similar results. In the two most distal channels, the mean difference was 9 Ω and 1 Ω, respectively. The Bland–Altman plot for MNBI-Z6 showed the distribution of points to be uniformly scattered, indicating a good agreement between the two methods and suggesting minimal bias when using either the simple or the conventional methods in this channel. However, the points outside the red dashed lines indicate discrepancies between the methods that may need further investigation.

There were certain limitations in our study. Firstly, the current software for MII-pH does not include an automated feature for measuring the conventional or simple MNBI. As a result, manual measurements of the MNBI were conducted, which are susceptible to human error. Artificial intelligence may be a promising solution for automatic measurement of the MNBI, according to recent studies. AI systems can provide automated analysis of MII-pH tracings, significantly enhancing the accuracy and efficiency of these measurements. With the capability of identifying the PSPW with an accuracy of up to 82%, AI technologies can streamline the diagnostic process by reducing human error and expediting the interpretation of complex data. As AI continues to evolve, its role in the clinical setting is expected to expand, offering even greater advancements in the diagnosis and management of GERD through the sophisticated analysis of impedance parameters [54,55,56]. Additionally, it should be noted that the study sample size was small, and patients were evaluated from a single tertiary center, potentially limiting the generalization of the findings to wider populations. These are preliminary results, and the study will be continued in order to provide more accurate data.

Further research is needed to determine the appropriate thresholds and to develop protocols for consistent measurements in future studies to be conducted across multiple centers.

## 5. Conclusions

Our study provides evidence for the value of utilizing simple MNBI measurement in the two distal channels to differentiate between different phenotypes of GERD in children. According to our findings and the available evidence, we highly encourage the use of MNBI as a routine method of assessing MII-pH monitoring in children who have negative upper endoscopy results and who show symptoms of GERD despite receiving medication.

## Figures and Tables

**Table 1:** General characteristics and pH–impedance measurements among the subgroups.

	Group 1 (n = 15)			Group 2 (n = 49) *				
	GERD (n = 10)	Normal (n = 5)	*p*-Value	NERD (n = 12)	RH (n = 20)	FH (n = 13)	Normal RI-NOS (n = 4)	*p*-Value
**Age (months/years) (mean ± SD)**	6 ± 3.27	8 ± 3.67	**0.302**	13.42 ± 2.27	13.4 ± 3.6	14.46 ± 2.07	12.75 ± 4.72	**0.69**
**Sex (male, %)**	40	40	**NS**	75	40	23.08	75	**0.03**
**Weight-for-length/BMI-for-age percentile (%)**	34 ± 31.9	33 ± 30.4	**NS**	49.9 ± 28.3	47.4 ± 30.7	51.4 ± 38.4	63.5 ± 40.84	**0.85**
**Acid exposure time (%, median, IQR)**	1.8 (1.2–2.5)	0.4 (0.3–0.6)	**0.04**	5.9 (5.28–7.18)	1.35 (0.5–2.5)	0.4 (0.3–1.9)	0.95 (0.7–1.15)	**<0.001**
**Total reflux episodes number (mean ± SD)**	69.5 ± 26.7	51.8 ± 19.6	**0.21**	94.7 ± 44.3	66.3 ± 45.4	32.8 ± 15	36 ± 29.5	**0.003**
**Acid reflux episode number**	33.2 ± 23.3	17.2 ± 15.1	**0.14**	76.3 ± 37.8	31.6 ± 22.2	18.9 ± 11.8	20.8 ± 11.4	**<0.001**
**Proximal reflux episodes**	42.9 ± 22.3	31 ± 14.4	**0.3**	53.4 ± 24	34 ± 27.1	15.8 ± 6.7	17.3 ± 15.02	**0.001**
**Mean acid clearance time (s) (median, IQR)**	76.5 (61.5–126.75)	32.0 (32.0–51.0)	**0.07**	120.6 ± 79.8	62.7 ± 46.7	53.8 ± 51.8	44.5 ± 30.3	**0.015**
**Bolus clearance time (s) (mean ± SD)**	15.5 ± 4.6	13.6 ± 2.3	**0.4**	12.3 ± 3.55	15.4 ± 5.9	12.5 ± 3.18	16.5 ± 6.76	**0.16**
**PSPW index (%)**	36.4 ± 12.9	52.9 ± 12.6	**0.03**	36.1 ± 13.9	47.6 ± 14.8	69.9 ± 14.4	65.4 ± 11.3	**<0.001**
**Conventional** **MNBI-Z1**	1559 ± 775	1411 ± 650	**0.72**	2327 ± 1057	2724 ± 959	2453 ± 585	1725 ± 1233	**0.23**
**Conventional** **MNBI-Z2**	1674 ± 661	1795 ± 506	**0.72**	2536 ± 1066	2492 ± 721	2497 ± 537	1748 ± 1182	**0.37**
**Conventional** **MNBI-Z3**	1892 ± 560	2209 ± 351	**0.27**	2796 ± 1152	2895 ± 1013	2912 ± 594	2180 ± 1313	**0.59**
**Conventional** **MNBI-Z4**	2170 ± 528	2197 ± 418	**0.92**	2388 ± 1038	2887 ± 995	2811 ± 623	2127 ± 1298	**0.108**
**Conventional** **MNBI-Z5**	2334 ± 752	2155 ± 576	**0.65**	1812 ± 875	2645 ± 984	3256 ± 613	1941 ± 1146	**0.001**
**Conventional** **MNBI-Z6**	2342 ± 911	2357 ± 426	**0.97**	1121 ± 773	2281 ± 890	2882 ± 526	1652 ± 1225	**<0.001**

NERD, non-erosive reflux disease; RH, reflux hypersensitivity; FH, functional heartburn; Normal RI-NOS, normal reflux index not otherwise specified; SD, standard deviation; BMI for age—percentile, Body-mass index for age—percentile; MNBI, mean nocturnal baseline impedance; PSPW, post-reflux swallow peristaltic wave; IQR. Interquartile; Z1–Z6, impedance channels 1–6. * Partial data previously published in a pilot study [39].

**Table 2:** Simple method values of the mean nocturnal baseline impedance among groups.

	Group 1			Group 2				
	GERD (n = 10)	Normal (n = 5)	*p*-Value	NERD (n = 12)	RH (n = 20)	FH (n = 13)	Normal RI-NOS (n = 4)	*p*-Value
**Simple** **MNBI-Z1**	1731 ± 804	1526 ± 715	**0.63**	2300 ± 900	2697 ± 803	2469 ± 563	1803 ± 1244	**0.203**
**Simple** **MNBI-Z2**	1758 ± 578	1851 ± 335	**0.74**	2398 ± 946	2472 ± 634	2482 ± 605	1791 ± 1199	**0.751**
**Simple** **MNBI-Z3**	1883 ± 532	2180 ± 224	**0.25**	2641 ± 1089	2887 ± 995	2811 ± 623	2127 ± 1298	**0.520**
**Simple** **MNBI-Z4**	2177 ± 565	2062 ± 232	**0.68**	2257 ± 1044	2868 ± 1045	3136 ± 756	2084 ± 1276	**0.088**
**Simple** **MNBI-Z5**	2318 ± 760	2156 ± 538	**0.6**	1872 ± 853	2609 ± 976	3172 ± 600	2101 ± 1263	**0.005**
**Simple** **MNBI-Z6**	2259 ± 871	2342 ± 500	**0.84**	1194 ± 735	2233 ± 820	2868 ± 517	1708 ± 1128	**<0.001**

GERD, gastroesophageal reflux disease; NERD, non-erosive reflux disease; RH, reflux hypersensitivity; FH, functional heartburn; Normal RI-NOS, normal reflux index not otherwise specified; MNBI, mean nocturnal baseline impedance; Z1–Z6, impedance channels 1–6.

**Table 3:** Correlation between conventional and simple mean nocturnal baseline impedance values in all impedance channels in infants (Group 1).

	Absolute Values (Means)			Correlation	
	Conventional MNBI	Simple MNBI	*p*-Value	r-Value	*p*-Value
**MNBI-Z1**	1510	1663	0.873	0.95	<0.001
**MNBI-Z2**	1714	1789	0.320	0.88	<0.001
**MNBI-Z3**	1998	1982	0.143	0.93	<0.001
**MNBI-Z4**	2179	2138	<0.001	0.88	<0.001
**MNBI-Z5**	2274	2264	0.806	0.98	<0.001
**MNBI-Z6**	2347	2287	0.972	0.96	<0.001

MNBI, mean nocturnal baseline impedance; Z1–Z6, impedance channels 1–6.

**Table 4:** Correlation between conventional and simple mean nocturnal baseline impedance values in all impedance channels in children (Group 2).

	Absolute Values (Means)			Correlation	
	Conventional MNBI	Simple MNBI	*p*-Value	r-Value	*p*-Value
**MNBI-Z1**	2473	2466	0.873	0.95	<0.001
**MNBI-Z2**	2444	2401	0.320	0.93	<0.001
**MNBI-Z3**	2817	2744	0.143	0.94	<0.001
**MNBI-Z4**	2840	2725	<0.001	0.98	<0.001
**MNBI-Z5**	2545	2536	0.806	0.97	<0.001
**MNBI-Z6**	2105	2104	0.972	0.97	<0.001

MNBI, mean nocturnal baseline impedance; Z1–Z6, impedance channels 1–6.

**Table 5:** Correlations with the simple mean nocturnal baseline.

MNBI Impedance Channel	GERD Phenotype	Correlation Coefficient (r-Value)	*p*-Value
MNBI-Z5	NERD	0.952	<0.001
	RH	0.980	<0.001
	FH	0.897	<0.001
	Normal RI-NOS	0.4	0.6
MNBI-Z6	NERD	0.953	<0.001
	RH	0.973	<0.001
	FH	0.816	<0.001
	Normal RI-NOS	0.969	<0.001

NERD, non-erosive reflux disease; RH, reflux hypersensitivity; FH, functional heartburn; Normal RI-NOS, normal reflux index not otherwise specified; MNBI-Z5, mean nocturnal baseline impedance in the fifth impedance channel; MNBI-Z6, mean nocturnal baseline impedance in the most distal impedance channel.

## Data Availability

The data presented in this study are available on request from the corresponding author. The data are not publicly available due to privacy and ethical restrictions.
